# Association Between Social Jetlag and Low Back Pain in Brazilian Women

**DOI:** 10.7759/cureus.101516

**Published:** 2026-01-14

**Authors:** Lidiane Barazzetti, Anderson Garcez, Raquel Canuto, Patrícia Cilene Freitas Sant'Anna, Juvenal Soares Dias da Costa, Vera Maria Vieira Paniz, Maria Teresa Anselmo Olinto

**Affiliations:** 1 Post-graduate Program in Collective Health, University of Vale do Rio dos Sinos (UNISINOS), São Leopoldo, BRA; 2 Post-graduate Program in Medical Sciences: Endocrinology, Federal University of Rio Grande do Sul (UFRGS), Porto Alegre, BRA; 3 Post-graduate Program in Food, Nutrition and Health, Federal University of Rio Grande do Sul (UFRGS), Porto Alegre, BRA

**Keywords:** chronic low back pain, sleep, social jetlag, women, women's health

## Abstract

Introduction

Chronic low back pain (CLBP) is one of the biggest public health issues globally and can have a major impact on an individual’s quality of life. Social jetlag (SJL) comprises a misalignment in an individual's circadian rhythm due to social imposition. Previous studies have mentioned a possible relationship between sleep-related disorders and the presence of CLBP.

Objectives

The present study aimed to explore the association between SJL and CLBP in women.

Methodology

A cross-sectional population-based study was conducted with a representative sample of 1068 women aged between 20 and 69 years from Southern Brazil. The presence of SJL was evaluated according to the absolute difference between the onset of sleep on free days and the onset of sleep on working days. CLBP was considered present if the pain persisted for ≥ 12 weeks. Poisson regression with robust variance was used to estimate prevalence ratios (PRs) with a 95% confidence interval (CI).

Results

The prevalence of CLBP in the sample population was 46.8% (95% CI: 43.8-49.8). The prevalence of SJL of <1 hour was 68.3% and that of SJL of ≥ 1 hour was 31.5% (16.6% with one to two hours and 14.9% with ≥ 2 hours). After stratification of the sample for use of prescription pain medications, women with SJL of ≥ 2 hours had an 83% higher probability of having CLBP when compared to women with a SJL of < 1 hour (PR = 1.83; 95% CI: 1.40-2.40; p < 0.001). This included adjustment for potential confounding factors.

Conclusions

The findings of this study revealed that women who used prescription pain medications and had SJL of ≥ 2 hours had an 83% higher probability of having CLBP. The use of prescription pain medications has been shown to be an interaction factor in the relationship between SJL and CLBP.

## Introduction

Chronic low back pain (CLBP) is defined as pain, muscle tension, or discomfort located below the costal margin and above the lower edge of the buttocks, with or without sciatica, for a minimum period of three consecutive months [1]. It is one of the biggest public health issues globally and can have a major impact on an individual’s quality of life. It is also correlated with high rates of work disability in individuals. CLBP affects women more than men, particularly those of economically active age, accounting for 60.1 million of all health treatment cases worldwide in 2015 [2].

Previous studies have reported an association between sleep-related disorders and the presence of CLBP [3], even considering these disorders as possible moderators of the association of CLBP in women with other health conditions, such as common mental disorders [4]. Since sleep health has emerged as a multidimensional construct comprising several aspects, such as quality and intensity, the concept of social jetlag (SJL) emerged to describe the restriction/extension of sleep duration when comparing days that included social obligations (weekdays) to free days (weekends) [5].

SJL comprises a misalignment in an individual's circadian rhythm due to social imposition. These misalignments represent an abnormal difference between two or more rhythms, whether internal or external [6]. Originally, SJL was defined as the absolute difference between the average point of sleep on free days and the sleep midpoint on working days [6]. Recently, Roenneberg and colleagues proposed the corrected SJL, which considers the effects of accumulated sleep debt on weekdays [7].

Previous studies have mentioned a possible relationship between SJL and chronic health conditions, such as metabolic syndrome, or with certain parameters of the metabolic syndrome [8,9]. Its association with cardiovascular diseases [10] and inflammatory bowel disease [11] is controversial, and its association with chronic musculoskeletal diseases such as CLBP remains unelucidated.

This study aimed to examine the association between SJL and CLBP in a population-based sample of women aged 20-69 years. We hypothesized a priori that women experiencing SJL of ≥ 2 hours would have a higher prevalence of CLBP. Additionally, we hypothesized that this association could be modified by health-related variables, including the use of prescription pain medications. The focus on women within this age range was justified by evidence indicating that CLBP affects women more frequently than men, particularly during economically active ages.

## Materials and methods

Study design and population

This investigation employed a population-based, cross-sectional observational design with a representative sample of women aged 20 to 69 years residing in the urban area of São Leopoldo, Rio Grande do Sul, Brazil. Data were derived from a broader research project entitled “Living Conditions and Health of Adult Women: A Population-Based Study in Vale dos Sinos - Ten-Year Follow-Up Evaluation,” conducted in 2015. Detailed information regarding sample size determination and additional methodological procedures of the parent study has been previously published [12]. The study protocol was reviewed and approved by the Research Ethics Committee of UNISINOS (approval number 653.394), and written informed consent was obtained from all participants prior to enrollment.

Sample and sampling

Sampling was conducted using a cluster design across 45 census tracts that had been previously selected. Within each cluster, a residential block was randomly chosen as the starting point, and the initial household for data collection was defined based on a predetermined street corner. All women aged 20 to 69 years residing in the selected households were invited to participate. Exclusion criteria included a history of lumbar spine fracture or lumbar spine surgery within the preceding six months, current pregnancy, and absence from the household at the time of data collection. Based on the sample size of the parent study (n = 1,068), a post hoc statistical power analysis was performed to assess the association between SJL and CLBP, adjusting for potential confounders. This sample size provided 95% confidence, 80% statistical power, and the ability to detect a prevalence ratio (PR) of 1.18 for the association between SJL and CLBP. Data were collected using standardized, pre-coded, and previously pilot-tested questionnaires encompassing demographic, socioeconomic, and behavioral variables. Anthropometric measurements, including body weight, height, and waist circumference, were obtained following standardized procedures. To ensure data reliability, 10% of the sample was randomly selected for repeat interviews, conducted either face-to-face or by telephone. Data collection took place between February and October 2015.

Chronic low back pain - outcome

CLBP was defined as low back pain persisting for a duration of at least 12 weeks (three months). A response card with an adapted illustration was used to identify the lumbar region during the data collection [13]. This card was developed based on the Nordic Questionnaire [13], a validated instrument created by Kuorinka et al. [13] in 1987 to determine the presence of musculoskeletal issues in specific anatomical regions of the body, including the lower back. After identifying the lumbar region, participants were first asked whether they had experienced pain in the lumbar region during the last two weeks. Participants who responded affirmatively were subsequently asked about the total duration of their lumbar pain. Participants reporting a pain duration of at least 12 weeks were classified as having CLBP, which was assessed dichotomously (yes/no). Although the lumbar region was queried, CLBP was considered non-specific pain located at or just below the waist, with or without associated buttock pain.

Social jetlag - main exposure

SJL is considered the restriction/extension of sleep duration on weekdays when compared to free days that do not involve social obligations (weekend). To construct the SJL variable, two questions were asked regarding the participants' sleep habits during the last month: "during the last month, what time did you lay/go to bed at night?" This question asked about the usual time of lying down on weekdays, as well as the usual bedtime on weekends. Participants were also asked about the time (in minutes) that they took to fall asleep on both weekdays and weekends. Corrected SJL was calculated according to the method proposed by Roenneberg et al. [7]. Briefly, sleep onset was defined as bedtime plus sleep latency, and mid-sleep times were calculated separately for workdays and free days as the midpoint between sleep onset and wake-up time. To account for potential sleep debt accumulated on workdays, the mid-sleep time on free days was corrected by subtracting half of the difference between free-day sleep duration and the average weekly sleep duration. SJL was then defined as the absolute difference between the corrected mid-sleep on free days and the mid-sleep on workdays. SJL was categorized into < 1 hour (low SJL), ≥ 1 hour and < 2 hours (medium SJL), and ≥ 2 hours (high SJL) based on the cutoff points previously stipulated in the literature to investigate associations between SJL and health outcomes [14,15].

Covariates

Demographic, socioeconomic, behavioral, anthropometric, reproductive, and health-related variables were considered to characterize the study population and to control for potential confounding factors in the multivariable analyses. Demographic variables included age, recorded in completed years and categorized into five groups (20-29, 30-39, 40-49, 50-59, and 60-69 years); self-reported skin color, classified as White or non-White (Black, Brown, Yellow, or Indigenous); and marital status, categorized as living with a partner (married) or without a partner (single, separated, divorced, or widowed). Socioeconomic variables comprised years of formal education (≤4, 5-7, 8-10, 11-14, and ≥15 years), per capita household income expressed in minimum wages (<1, 1-3, 3.1-6, and >6 minimum wages), based on the Brazilian minimum wage in January 2015 (R$ 788.00), and work schedule, classified according to the presence or absence of a night or rotating shift. An inverted work shift was defined as having worked at least one night shift in the current or most recent employment. Behavioral variables included smoking status (never smoker, current smoker, or former smoker), alcohol consumption (abstinent, low/moderate intake (<15 g of alcohol/day), or high intake (≥15 g/day)), physical activity level, and sleep duration. Physical activity was assessed using the short version of the International Physical Activity Questionnaire (IPAQ) validated for use in Portuguese; participants reporting ≥150 minutes per week of moderate-to-vigorous physical activity were classified as physically active. Sleep duration was calculated as the average daily number of hours slept on weekdays and weekends and categorized as <6 hours, six to eight hours, or >8 hours per day. Anthropometric assessment focused on abdominal obesity, evaluated by waist circumference. Measurements were obtained at the midpoint between the lowest rib margin and the iliac crest using a non-elastic measuring tape. Waist circumference was measured in duplicate, and the mean value was used for analysis. Abdominal obesity was defined as a waist circumference ≥80 cm, and abdominal obesity level II as ≥88 cm, in accordance with established cutoff points [16]. All anthropometric measurements were performed by trained interviewers. Reproductive and health-related variables included menopausal status, symptoms of common mental disorders, and the use of prescription analgesic medications. Menopausal status was classified as premenopausal (regular menstrual cycles), perimenopausal (irregular menstrual cycles), or postmenopausal (absence of menstruation for at least 12 consecutive months). Symptoms of common mental disorders were assessed using the validated Self-Reporting Questionnaire (SRQ-20), which screens for symptoms of depression, anxiety, insomnia, fatigue, irritability, forgetfulness, difficulty concentrating, and somatic complaints over the previous 30 days [17]. The SRQ-20 consists of 20 dichotomous (yes/no) items, with a score of ≥8 affirmative responses indicating the presence of common mental disorder symptoms [17]. The use of prescribed medication for chronic pain was evaluated based on the identification and use of medications prescribed by a physician that the participant reported through the question, "Are you (Mrs.) currently using any medication prescribed by a doctor?" For the correct registration of each drug, a presentation of the prescription, packaging, or package leaflet was requested. To determine the health condition for which each drug was used, consideration was given to: (1) the main therapeutic indication of the drug (established by the Anatomical Therapeutic Chemical (ATC) classification [18], a system adopted by the World Health Organization to classify medicines according to the organ or system in which they operate) (2) the indication of use reported by the patient, and (3) other medicines used and their therapeutic indication. Those who reported use of prescription medications classified as N02 or M01 according to the ATC were classified as using medications for chronic pain (opioid and non-opioid analgesics and anti-inflammatory drugs).

Statistical analysis

Data were entered into EpiData software (version 3.1, Jens M. Lauritsen, Odense, Denmark) using a double-entry procedure, followed by validation through comparison and resolution of discrepancies. Stata software (version 12.0; Stata Corporation, College Station, TX) was used to identify potential database inconsistencies and to perform all statistical analyses. Descriptive analyses were conducted to characterize the study population, with absolute and relative frequencies used to describe the sample and to estimate the prevalence of the outcome (CLBP) and the main exposure (SJL), as well as their distribution across demographic, socioeconomic, behavioral, anthropometric, and health-related covariates. Bivariate analyses were performed using the chi-square test for heterogeneity for nominal and categorical variables and the chi-square test for linear trend for ordinal variables to assess associations between the outcome and the main exposure across covariate categories. Poisson regression models with robust variance were applied to estimate crude and adjusted PRs and their respective 95% confidence intervals (95% CIs) for the association between SJL and CLBP. A hierarchical modeling strategy defined a priori was adopted for multivariable adjustment [19], comprising the following models: Model I (unadjusted); Model II (adjusted for demographic and socioeconomic variables); Model III (adjusted for demographic, socioeconomic, behavioral, anthropometric, reproductive, and health-related variables); and Model IV (adjusted for demographic, socioeconomic, behavioral, anthropometric, reproductive, health-related variables, and use of prescription analgesics). Variables associated with the outcome or the main exposure at a p-value < 0.10 were considered potential confounders and included in the multivariable analyses. In addition, health-related variables (abdominal obesity, symptoms of common mental disorders, and use of prescription pain medications) were evaluated as potential effect modifiers in the association between SJL and CLBP using stratified analyses with the Mantel-Haenszel test (M-H test). Associations with p-values < 0.05 were considered statistically significant.

## Results

Of the 1,281 women initially selected for participation, 153 (11.9%) were classified as losses or refusals. A total of 1,128 women were interviewed; however, 60 participants (5.3%) presented data inconsistencies and were excluded. Consequently, the final analytical sample comprised 1,068 women aged 20 to 69 years, with a mean age of 43.2 ± 13.3 years.

Table 1 presents the distribution of the general characteristics of the sample population. Most women were over 40 years of age, lived with a partner, had white skin color, had a per capita family income of up to three minimum wages, were non-smokers, consumed alcohol mildly to moderately, were considered physically inactive, and most slept six hours or fewer a day. It was also observed that 1/3 of the women were in postmenopausal status, 2/3 had common mental disorders symptoms, approximately 50% had abdominal obesity, and approximately 10% used prescription pain medications.

**Table 1:** Sample characteristics and distribution of the prevalence of chronic low back pain according to demographic, socioeconomic, behavioral, anthropometric, and health characteristics in women from Southern Brazil, 2015 (n = 1068). MW, minimum wage; PC, per capita; WC, Waist Circumference; SRQ-20, Self-Report Questionnaire ^*^Linear trend test (used for ordinal variables). ^**^Chi-square test for heterogeneity of proportions (used for categorical and nominal variables). ^#^Social jetlag: absolute difference between the onset of sleep on free days and the onset of sleep on working days. Analyses were performed using all available data; the number of observations varied for the inverted work shift variable due to missing information.

Characteristics	n (%)	Chronic low back pain		p-value
		No	Yes	
		n (%)	n (%)	
Age (years)				<0.001*
20-29	206 (19.3)	142 (68.9)	64 (31.1)	-
30-39	234 (21.9)	133 (56.8)	101 (43.2)	-
40-49	260 (24.3)	117 (45.0)	143 (55.0)	-
50-59	217 (20.3)	101 (46.5)	116 (53.5)	-
60-69	151 (14.1)	75 (49.7)	76 (50.3)	-
Skin color				0.690**
White	790 (74.0)	423 (53.5)	367 (46.5)	-
Others	278 (26.0)	145 (52.2)	133 (47.8)	-
Marital status				0.002**
With a partner	383 (35.9)	136 (63.0)	166 (43.3)	-
Without a partner	685 (64.1)	351 (51.2)	334 (48.8)	-
Schooling (years of study)				<0.001
≤4	190 (17.8)	86 (45.3)	104 (54.7)	-
5-7	246 (23.0)	121 (49.2)	125 (50.8)	-
8-10	188 (17.6)	91 (48.4)	97 (51.6)	-
11-14	340 (31.8)	200 (58.8)	140 (41.2)	-
≥15	104 (9.7)	70 (53.2)	34 (37.2)	-
PC family income in MW				0.005*
<1	480 (44.9)	237 (49.4)	243 (50.6)	-
1-3	462 (43.3)	251 (54.3)	211 (45.7)	-
3.1-6	94 (8.8)	60 (63.8)	34 (36.2)	-
>6	32 (3.0)	20 (62.5)	12 (37.5)	-
Inverted work shift (n = 1037)				0.105**
No	970 (90.8)	519 (53.5)	451 (46.5)	-
Yes	67 (6.3)	29 (43.3)	38 (56.7)	-
Smoking				0.292**
Non-smoker	627 (58.7)	346 (55.2)	281 (44.8)	-
Former smoker	242 (22.7)	121 (50.0)	121 (50.0)	-
Smoking	199 (18.6)	101 (50.8)	98 (49.2)	-
Alcohol consumption				0.943
Abstinent	419 (39.2)	221 (52.7)	198 (47.3)	-
Light and moderate (<15 g/day)	569 (53.3)	307 (54.0)	262 (46.0)	-
High (≥15 g/day)	80 (7.5)	40 (50.0)	40 (50.0)	-
Physical activity				0.004**
Active	157 (14.7)	100 (63.7)	57 (36.3)	-
Inactive	911 (85.3)	468 (51.4)	443 (48.6)	-
Sleep duration (hours/day)				0.626*
< 6	99 (9.3)	52 (52.5)	47 (47.5)	-
6-8	526 (49.2)	276 (52.5)	250 (47.5)	-
> 8	443 (41.5)	240 (54.2)	203 (45.8)	-
Abdominal obesity				<0.001*
No (WC < 79.9cm)	296 (27.7)	183 (61.8)	113 (38.2)	-
Level I (WC 80-87.9cm)	246 (23.0)	134 (54.5)	112 (45.5)	-
Level II (WC ≥ 88cm)	526 (49.3)	251 (47.7)	275 (52.3)	-
Menopausal status				0.001*
Premenopausal	609 (57.0)	354 (58.1)	255 (41.9)	-
Perimenopausal	86 (8.1)	34 (39.5)	52 (60.5)	-
Postmenopausal	373 (34.9)	180 (48.3)	193 (51.7)	-
Common mental disorders symptoms				<0.001**
No (SRQ-20 < 8)	710 (66.4)	430 (60.6)	280 (39.4)	-
Yes (SRQ-20 ≥ 8)	358 (33.5)	138 (38.5)	220 (61.5)	-
Use of prescription pain medications				<0.001**
No	971 (90.6)	532 (54.7)	439 (45.2)	-
Yes	97 (9.4)	36 (37.1)	61 (62.9)	-
Social jetlag^#^				0.544*
<1 hour	730 (68.4)	394 (54.0)	336 (46.0)	-
≥1 hour and <2 hours	179 (16.8)	91 (50.8)	88 (49.2)	-
≤2 hours	159 (14.9)	83 (52.2)	76 (47.8)	-

The prevalence of CLBP was n=500 (46.8%; 95% CI:43.8-49.8). It was more prevalent in women >40 years, those with less schooling, lower per capita family income, working on the reverse shift (night shift), smokers or former smokers, those with high alcohol consumption, and physical inactivity. It was also more prevalent in women in the peri-/post-menopausal period, women with common mental disorder symptoms, abdominal obesity, and those who used prescription pain medications (Table 1). Among the women in the sample, 730 (68.3%) had SJL of < 1 hour, 338 (31.5%) had SJL ≥ 1 hour, including 179 (16.6%) between one and two hours, and 159 (14.9%) with ≥ 2 hours. Table 2 shows the distribution of SJL occurrence according to the sample characteristics. It was observed that SJL was more prevalent in younger, single women, with higher schooling, and in those with greater alcohol consumption, in the pre-menopausal period, and without abdominal obesity.

**Table 2:** Distribution of categories of social jetlag according to demographic, socioeconomic, behavioral, anthropometric, and health characteristics in women from Southern Brazil, 2015 (n = 1068). MW, minimum wages; PC, per capita; WC, Waist Circumference; SRQ-20, Self-Report Questionnaire ^*^Linear trend test (used for ordinal variables). ^**^Chi-square test for heterogeneity of proportions (used for categorical and nominal variables). ^#^Social jetlag: absolute difference between the onset of sleep on free days and the onset of sleep on working days. Analyses were performed using all available data; the number of observations varied for the inverted work shift variable due to missing information.

Characteristics	Social jetlag^#^			p-value
	<1 hour	≥1 hour and <2 hours	≥2 hours	
	n (%)	n (%)	n (%)	
Age (years)				<0.001*
20-29	107 (51.9)	45 (21.8)	54 (26.2)	-
30-39	154 (65.8)	44 (18.8)	36 (15.4)	-
40-49	170 (65.4)	53 (20.4)	37 (14.2)	-
50-59	169 (77.9)	27 (12.4)	21 (9.7)	-
60-69	130 (86.1)	10 (06.6)	11 (7.3)	-
Skin color				0.466**
White	534 (67.6)	139 (17.6)	117 (14.8)	-
Others	196 (70.5)	40 (14.4)	42 (15.1)	-
Marital status				0.036**
With a partner	247 (64.5)	65 (17.0)	71 (18.5)	-
Without a partner	483 (70.5)	114 (16.6)	88 (12.8)	-
Schooling (years of study)				0.003*
≤4	141 (74.2)	27 (14.2)	22 (11.6)	-
5-7	187 (76.0)	30 (12.2)	29 (11.8)	-
8-10	124 (66.0)	28 (14.9)	36 (19.1)	-
11-14	212 (62.4)	68 (20.0)	60 (17.6)	-
≥15	66 (63.5)	26 (25)	12 (11.5)	-
PC family income in MW				0.405*
<1	322 (67.1)	80 (16.7)	78 (16.3)	-
1-3	321 (69.5)	74 (16.0)	67 (14.5)	-
3.1-6	67 (71.3)	19 (20.2)	8 (8.5)	-
>6	20 (62.5)	6 (18.8)	6 (18.8)	-
Inverted work shift (n = 1037)				0.115**
No	657 (67.7)	170 (17.5)	143 (14.7)	-
Yes	47 (70.1)	6 (9.0)	14 (20.9)	-
Smoking				0.355**
Non-smoker	424 (67.6)	112 (17.9)	91 (14.5)	-
Former smoker	176 (72.7)	34 (14.0)	32 (13.2)	-
Smoking	130 (65.3)	33 (16.6)	36 (18.1)	-
Alcohol consumption				<0.001**
Abstinent	321 (76.6)	59 (14.1)	39 (9.3)	-
Light and moderate (<15 g/day)	369 (64.9)	106 (18.6)	94 (16.5)	-
High (≥15 g/day)	40 (50.0)	14 (17.5)	26 (32.5)	-
Physical activity				0.223**
Active	98 (62.4)	31 (19.7)	28 (17.8)	-
Inactive	632 (69.4)	148 (16.2)	131 (14.4)	-
Sleep duration (hours/day)				0.646*
<6	70 (70.7)	15 (15.2)	14 (14.1)	-
6-8	361 (68.6)	83 (15.8)	82 (15.6)	-
>8	299 (67.5)	81 (18.3)	63 (14.2)	-
Abdominal obesity				0.032*
No (WC < 79.9cm)	187 (63.2)	51 (17.2)	58 (19.6)	-
Level I (WC 80-87.9cm)	176 (71.5)	40 (16.3)	30 (12.2)	-
Level II (WC ≥ 88cm)	367 (69.8)	88 (16.7)	71 (13.5)	-
Menopausal status				<0.001*
Pre-menopause	372 (61.1)	122 (20.0)	115 (18.9)	-
Peri-menopause	58 (67.4)	18 (20.9)	10 (11.6)	-
Post-menopause	300 (80.4)	39 (10.5)	34 (9.1)	-
Common mental disorders symptoms				0.349**
No (SRQ-20 < 8)	481 (67.7)	127 (17.9)	102 (14.4)	-
Yes (SRQ-20 ≥ 8)	249 (69.6)	52 (14.5)	57 (15.9)	-
Use of prescription pain medications				0.392**
No	659 (67.8)	163 (16.8)	149 (15.3)	-
Yes	71 (73.2)	16 (16.5)	10 (10.3)	-

Among the health covariates explored, abdominal obesity (M-H test p-value = 0.852) and symptoms of common mental disorders (M-H test p-value = 0.746) did not show a significant interaction with the relationship between SJL and CLBP; only the use of prescription pain medications exhibited a significant interaction in this relationship (M-H test p-value = 0.029). This justified the sample stratification by use of prescription pain medications. Table 3 lists the unadjusted and adjusted PRs for the association between SJL and CLBP in the total sample and stratified by the use of prescription pain medications. After stratification, a statistically significant association between SJL and CLBP was observed in women who used prescription pain medications. Moreover, it was observed that the higher the SJL, the higher the probability of CLBP; that is, there was a dose-response relationship, even after adjustment for potential confounding factors in the three multivariate models (II, III, and IV). Women with SJL of ≥ 2 hours had an 83% higher probability of having CLBP than women with a SJL of <1 hour (PR = 1.83; 95% CI: 1.40-2.40; p < 0.001). It is noteworthy that this relationship between SJL and CLBP was not observed in women who did not use prescription pain medications (Table 3).

**Table 3:** Unadjusted and adjusted prevalence ratios (PR) for the association between social jetlag# and chronic low back pain, in the total sample and stratified by use of prescribed medication for chronic pain, in women from Southern Brazil, 2015 (N = 1068). ^*^p-value for the Wald test for linear trend obtained by Poisson regression with robust variance. ^#^Social jet lag: absolute difference between the onset of sleep on free days and the onset of sleep on working days. Model: unadjusted analysis (without adjustment); Model II: adjusted for sociodemographic characteristics (age, marital status, schooling, and inverted work shift); Model III: adjusted for sociodemographic, behavioral, anthropometric, reproductive, and health variables (age, marital status, schooling and inverted work shift, alcohol consumption, physical activity, abdominal obesity, menopausal status, and common mental disorders symptoms); Model IV: adjusted for sociodemographic, behavioral, anthropometric, reproductive, and health variables (age, marital status, schooling, inverted work shift, alcohol consumption, physical activity, abdominal obesity, menopausal status, common mental disorders symptoms, and use of prescription pain medications). Mantel-Haenszel test: interaction test of the variable 'use of prescription pain medications' in the association between social jetlag and CLBP (p = 0.029).

Exposure variable	Chronic low back pain			
	Model I	Model II	Model III	Model IV
	PR (95% CI)	PR (95% CI)	PR (95% CI)	PR (95% CI)
Total sample				
Social jetlag				
<1 hour	1.00	1.00	1.00	1.00
≥1 hour and <2 hours	1.06 (0.90-1.26)	1.16 (0.98-1.37)	1.16 (0.99-1.37)	1.16 (0.98-1.36)
≥2 hours	1.03 (0.86-1.24)	1.14 (0.95-1.36)	1.11 (0.93-1.32)	1.11 (0.93-1.31)
p-value*	0.539	0.060	0.106	0.109
No use of prescription pain medications				
Social jetlag				
<1 hour	1.00	1.00	1.00	-
≥1 hour and <2 hours	1.05 (0.85-1.26)	1.14 (0.94-1.37)	1.15 (0.96-1.38)	-
≥2 hours	0.98 (0.80-1.20)	1.08 (0.88-1.31)	1.06 (0.87-1.28)	-
p-value*	0.960	0.257	0.330	-
Using prescription pain medications				
Social jetlag				
<1 hour	1.00	1.00	1.00	-
≥1 hours and <2 hours	1.22 (0.85-1.80)	1.21 (0.85-1.74)	1.16 (0.84-1.62)	-
≥2 hours	1.77 (1.44-2.18)	1.81 (1.40-2.32)	1.83 (1.40-2.40)	-
p-value*	<0.001	<0.001	<0.001	-

## Discussion

This study examined the association between SJL and CLBP in a sample of women. A high prevalence of CLBP was found, and approximately 1/3 of the sample population had SJL for at least one hour. Although there was no relationship between SJL and CLBP in the sample as a whole, among women who used prescription pain medications, this relationship presented a highly significant association. It was observed that the higher the SJL, the greater the probability of having CLBP was.

In this study, more than 50% of women over 50 years of age presented with CLBP. A high prevalence of CLBP has also been reported in similar populations, especially in low- and middle-income countries [20] and Latin American populations [21]. Regarding the main exposure, 14.9% of women had SJL of ≥ 2 hours. The distribution of SJL in the population studied followed patterns already described by another population-based study by the Korean Ministry of Health and Welfare, conducted with Korean adults [22], and another study by the Furukawa Nutrition and Health Study, conducted with Japanese workers [14].

Regarding epidemiology (i.e., the distribution of these two events in the sample), characteristics that could be considered divergent were observed. CLBP was more prevalent in women >40 years, those who were married or living with a partner, those with lower schooling, lower income, physical inactivity, in the peri/post-menopausal period, and disadvantaged health conditions such as the presence of abdominal obesity, common mental disorders symptoms, and use of prescription pain medications. It is noteworthy that the association of these characteristics with CLBP has already been described in the literature [23-25]. In contrast, SJL was more prevalent in younger women, those who were single or without a partner, those with good schooling, high alcohol consumption, and in women in the pre-menopausal period, and without abdominal obesity. Hence, SJL has proven to be a characteristic of the group of women entering adulthood. Similar characteristics have been found in other previously mentioned studies [14,22] with similar populations; however, no studies have explored SJL exclusively in female populations.

When studying the relationship between SJL and CLBP in women, our data did not identify an association when considering the sample as a whole. This finding was expected, since these events tend to occur in different and/or consecutive stages of women’s lives, particularly in women belonging to the age group studied (20-69 years). This was considered when evaluating the distribution of these two events according to the characteristics of the sample of women, as presented above. However, when investigating potential variables that could interact with SJL and CLBP, the use of prescription pain medications was identified as an interaction factor in this association. Thus, our findings showed a highly significant relationship between SJL and CLBP in women using prescription pain medications. Those with SJL of ≥ 2 hours were 83% more likely to have CLBP.

After an extensive review of the scientific literature, we found that no studies have investigated the association between SJL and CLBP or explored the potential interactions in the relationship between SJL and musculoskeletal diseases. Nevertheless, it is known that individuals with more disabling low back pain often use pain medications, such as analgesics or anti-inflammatory drugs [26]. It is possible that the women in this study presented with more severe and painful conditions, which would justify the use of prescription pain medications and may also be related to metabolic disorders, since the increase in inflammatory mediators is common in these situations. Some studies have already pointed to an association between SJL and an increased risk of cardiovascular disease [15], obesity, weight gain, and metabolic disorders [27,28]. SJL would subsequently have a stressful effect on the human body since it is a chronic condition. It is known that stress mechanisms promote excessive secretion of glucocorticoid hormones from the adrenal gland, especially cortisol, which is a biomarker of psychological stress. Cortisol production and release are also controlled by the circadian pacemaker located in the suprachiasmatic nucleus of the hypothalamus and by changes in the sleep-wake cycle [29,30].

Due to this stressful effect, individuals with an SJL of ≥2 hours would have higher cortisol levels during the day when compared to individuals with lower SJL [15]. In turn, increased cortisol levels may be related to an increase in inflammatory biomarkers in the body [31]. In the Dunedin Longitudinal Study, although the association between jetlag and inflammatory biomarkers was not significant, individuals with two-hour SJL had levels of inflammatory biomarkers approximately twice as high as those in individuals without SJL [27]. Thus, the women in this study possibly presented with two conditions related to the increase in inflammatory biomarkers in the body: the stressful effect imposed by the SJL and the use of prescription pain medications. This raises the hypothesis that the severity of pain could generate a reverse causal effect on the relationship between SJL and CLBP.

The findings of this study are scientifically relevant, as CLBP imposes a substantial economic burden on health care systems [1,2]. The characteristics of today's society, which reflect behavioral changes in habits that regulate the circadian cycle, can affect physical, social, and emotional health [6,7]. One example of these characteristics is SJL. Thus, establishing the relationship between SJL and CLBP can contribute to better health promotion and prevention actions that focus mainly on human habits and behaviors, as many of them are imposed by society, especially for the female population.

The literature regarding the association between SJL and chronic health conditions remains controversial. Some studies reported a positive association between SJL and chronic inflammatory bowel disease [11] and between SJL and the presence of severe menstrual symptoms [32]. In another study, the association between SJL and bruxism was statistically significant [33]. Notably, none of these studies sought to investigate possible interactions. To our knowledge, this is the first study to investigate the association between SJL and CLBP. A major strength of the study is its population-based design with a representative sample of women, as well as the use of previously tested and validated instruments to assess CLBP and symptoms of common mental disorders. Furthermore, for the evaluation of SJL presence, the use of SJL (which also corrects for sleep debt) stands out, since SJL arises from the differences between the sleep time on free days and working days, as well as the effects of accumulated sleep debt [7,34]. Therefore, the use of SJL helps avoid any potential bias in the results [35]. Of note, multivariate analyses were performed for the associations explored between SJL and CLBP, including controlling for important confounding factors and interaction evaluation for the explored variables, which reinforces the methodological rigor used in this study.

Limitations

This study had some limitations related to the design. In studies with a cross-sectional design, causal relationships between exposure and outcome cannot be established, and the possibility of reverse causality cannot be excluded. Furthermore, the presence of an association between SJL and CLBP only when the use of prescribed medication for chronic pain is present reinforces the idea that SJL could have a stressful effect and potentially trigger an increase in inflammatory mediators [15], particularly in individuals with more intense painful conditions who require prescribed medication for chronic pain. However, the use of prescription pain medication as a stratification variable may be subject to confounding by indication, as analgesic use may serve as a proxy for underlying pain severity and comorbidity burden; this should be carefully considered when interpreting the study findings. Information about bedtime and waking was reported by the study participants, and this may lead to a possible non-differential information bias. A limitation of this study is that the underlying causes of low back pain were not investigated. It is important to note that, as most cases of CLBP are classified as nonspecific and may originate from anatomical structures beyond the lumbar spine, such as the sacroiliac joints, the wording of the pain assessment used in this study (lumbar spine) may not have fully captured all potential sources of CLBP, potentially leading to misclassification. Another limitation is that information about chronic disease conditions that affect both sleep and chronic pain, such as fibromyalgia, rheumatoid arthritis, and others, was not collected and explored as covariates in multivariate analysis. The use of waist circumference as the sole anthropometric indicator represents a limitation, as it does not fully capture overall obesity, body composition, or the complexity of fat distribution. Our study was also limited in its ability to control for variability in behaviors associated with social jet lag, including delayed bedtimes due to social commitments, nighttime social activities, and weekend-related schedule shifts that result in later sleep onset. We recognize this as an important aspect to be investigated and explored in future studies. The external validity of this study is noteworthy, as it was conducted in a representative sample of adult women residing in a city in southern Brazil. Finally, it is worth noting that the association between SJL and CLBP may be bidirectional, with the relationship proposed by this study between these variables and the use of prescribed pain medication also possibly occurring in other ways. For instance, CLBP could predict the use of analgesics, and in turn, these medications could predict sleep disturbances. Therefore, further investigation into this relationship requires more studies, especially longitudinal studies that can establish a causal relationship between these variables. Future research would benefit from repeated assessments of pain intensity following periods of social jet lag compared with periods of adequate sleep, as well as from the inclusion of more detailed clinical or biomechanical evaluations, such as assessments of sacroiliac joint involvement or pelvic alignment, to help clarify potential pain mechanisms and anatomical origins.

## Conclusions

The findings of this study revealed that women who used prescribed medication for chronic pain and who had SJL of ≥2 hours were 83% more likely to have CLBP. The use of prescription pain medications was shown to be an interaction factor in the relationship between SJL and CLBP. Nevertheless, further research is warranted to examine this association, particularly population-based and longitudinal studies that may contribute to establishing a causal relationship between these variables. It is plausible that sleep deprivation exacerbates pain severity, thereby increasing the need for pain medication, rather than analgesic use constituting a primary cause of low back pain.
